# The need for additional mental health support for women in the postpartum period in the times of epidemic crisis

**DOI:** 10.1186/s12884-021-03544-8

**Published:** 2021-02-08

**Authors:** Magdalena Chrzan-Dętkoś, Tamara Walczak-Kozłowska, Małgorzata Lipowska

**Affiliations:** 1 Institute of Psychology, University of Gdansk, Jana Bażyńskiego 8, 80-309, Gdańsk, Poland; 2 Department of Psychology, Gdansk University of Physical Education and Sport, Kazimierza Górskiego 1, 80-336, Gdańsk, Poland

**Keywords:** Epidemic crisis, Postpartum depression, Motherhood, Affective disorder, Edinburgh postnatal depression scale, Covid-19

## Abstract

**Background:**

This retrospective study aimed to identify possible intensification of mental health difficulties among women seeking support in the postpartum period during the epidemic state in Poland. We assumed that the epidemic crisis, social isolation, and restrictions in hospitals which affect pregnant and postpartum women - lack of family labors, lack of the possibility to be with the newborn when he/she is hospitalized, may increase fear and reduce psychosocial resources of women, hinder their normal process of transition to motherhood and thus contribute to the intensified severity of depressive symptoms.

**Methods:**

The study participants were women seeking support at the on-line platform of the project ‘Next Stop: Mum’, which is a part of the postpartum depression prevention’s program implemented by the Ministry of Health in Poland, and enables remote self-screening for the severity of the postpartum depression symptoms with the Edinburgh Postnatal Depression Scale developed by Cox and collaborators. The analyzed data in this study were obtained from 139 women: 61 filled forms from October 1 - November 10, 2019 (non-epidemic period), and 78 filled forms from February 20–March 30 (beginning of the COVID-19 epidemic), 2020.

**Results:**

A statistically significant difference in the severity of postpartum depression symptoms were observed among women making a self-assessment with EPDS scale at the beginning of the COVID-19 epidemic in Poland (M = 15.71; SD = 6.23), compared to the pre-epidemic neutral period (M = 13.56; SD = 6.46).

**Conclusions:**

The results of this study indicate that the epidemic crisis may be associated with an increased need for additional caution and support of women’s mental health in the postpartum period. We believe that recommendations for medical staff, policy, and families of women struggling with postpartum depression symptoms during crisis should be widespread as the second wave of COVID-19 disease may develop in the autumn-winter 2020 and spring 2021.

**Supplementary Information:**

The online version contains supplementary material available at 10.1186/s12884-021-03544-8.

## Background

The global epidemic crisis is having a profound effect on all aspects of our life. In Poland, as well as in other European countries self-isolation and severe restriction in everyday routine were introduced in early March 2020. A study carried out in Poland 10 days after introducing restrictions [1] showed that the level of anxiety in Polish society was quite high e.g. 75% of the respondents were worried that some people would not follow the government’s instructions and the virus would spread too quickly, 73% were afraid of hospital overcrowding and healthcare system failure, 72% - losing loved ones, 71% - financial crisis and market collapse, and 70% - panic and irrational behaviour of other people. Moreover, 26% of Poles estimated that their anxiety reached the level of a panic attack. The study [1] also revealed that women felt greater fear in comparison to men at the beginning of the epidemic crisis.

According to international studies [2, 3], especially vulnerable to stress and mental health problems are a front-line health and social care staff, those with pre-existing health issues, young people (aged ≤18 years), and older adults (aged ≥65 years). However, beyond direct influence, the psychological and social effects of the COVID-19 epidemic are increasingly seen as pervasive factors that may affect mental health now and in the future [2]**.** Based on the studies concerning the early phase of the severe acute respiratory syndrome (SARS) outbreak, a range of psychiatric morbidities may be suspected including persistent depression, anxiety, panic attacks, psychomotor excitement, psychotic symptoms, delirium, and even suicidality [4–6]. Moreover, we believe that the COVID-19 epidemic crisis along with a high level of anxiety [1] could contribute to the intensification (or be a trigger) of mental health problems among those people who were at high-risk under normal (non-epidemic) conditions [7, 8]. We assumed that such factors as anxiety about the possibility of infection during pregnancy and/or after delivery, restrictions on delivery and hospital stay (limited contact with relatives and friends), limited access to specialist and control treatment (often restricted only to emergencies), a radical change in the postpartum care: midwife-woman relationship, where often the comforting presence, practical help in breastfeeding as the key supportive elements, has been transferred “online” in an attempt to maintain distance and reduce cross-infection, loss of social support due to voluntary quarantine and lockdown, confusion and panic (often increased by fake news) – all may affect the well-being and mental health of mothers in the postpartum. The existing psychosocial resources may not be sufficient to cope with the process of transition to motherhood. It is worth emphasizing that lack of social support is listed as one of the major risk factors for postpartum depression along with high life stress, current or past abuse, prenatal depression, and marital or partner dissatisfaction [8].

Previous studies [9, 10] described the effects of the COVID-19 epidemic on the depression and anxiety levels of pregnant women. A recent assessment of depression symptoms with the Edinburgh Postnatal Depression Scale revealed significantly higher rates of depressive symptoms among pregnant women assessed after the declaration of COVID-19 epidemic in comparison to women assessed in pre-epidemic period [10]. However, there are no reports on the occurrence of severe depressive symptoms among women in the postpartum period during an epidemic crisis.

Postpartum depression (PPD) is a common and serious mental health problem that affects about 13–20% of new mothers [11, 12]. In many cases PPD resolves spontaneously e.g. Whiteford et al. [13] reported the remission rate of 53% in adult samples experiencing depression within 1 year and O’Hara et al. [14] indicated that the symptoms last 7 months on average when left untreated. Yet still, about one in three women feel worse even more than a year after delivery, and research [15, 16] indicate that there are about 40% cases of relapses. Researchers [17–19] indicate that suicide accounts for one in five deaths and is the second leading cause of mortality in the first year postpartum. Therefore, screening procedures (to detect PPD symptoms) are widely implemented in many countries. Of course, screening test precedes the extended clinical examination but helps to quickly detect cases that may require fast professional help [20].

The severity of PPD symptoms is associated with many biological and non-biological factors e.g. Wisner et al. [19] indicate that the particular risk refers to those women with a personal or family history of depression, physical or sexual abuse, unplanned pregnancy, and pregnancy complications. There are also additional social factors that may influence depressive symptoms: for example, research indicates that shorter maternity leave is associated with greater depressive symptoms at 9 months postpartum [21, 22].

On the other hand, social support is an important protective factor [23]. However now, during the COVID-19 pandemic, new mothers are deprived of their social network. According to the British Academy of Medical Science, major adverse consequences of the epidemic crisis are increased social isolation and loneliness [24], which are strongly associated with anxiety, depression, self-harm, and suicide attempts across the lifespan [25, 26]. The aggravated depressive symptoms during the global epidemic crisis can thus be caused directly - by concerns about exposure to COVID-19 (an additional strong and widespread stressor) but also indirectly. Apart from the negative consequences of isolation, what was already mentioned, many families also encounter several changes in financial well-being and economic stability. Moreover, prolonged direct contact with other children (daily care and home education during a pandemic), change to home office for working mothers can intensify daily fatigue and stress, make work-life balance more difficult, trigger conflicts and interfere with adapting to life with a new baby. So, an epidemic crisis can limit a great part of the psychological resources that builds woman’s health in the postpartum period.

## Methods

### Aim and design

At the turn of February and March 2020, we began to observe an increase in the number of requests for psychological consultations via our on-line platform ‘Next Stop: Mum’ which offers mental health support for Polish women up to a year after giving birth. We began to wonder if this sudden increase in support requests from women may be associated with exacerbation of depressive symptoms caused by the outbreak of the COVID-19 epidemic in Europe. Thus, the purpose of this study was to characterize the mental state of women in the postpartum period seeking mental health support at the beginning of the epidemic crisis in Poland – late February and March 2020, when disturbing information about the virus began to spread to European countries, including Poland. It is worth adding that it was also the time of the greatest panic, confusion and the emergence of fake news.

To verify whether the severity of depressive symptoms was different at the beginning of the COVID-19 epidemic from the neutral (non-epidemic) period, we decided to compare the results of the severity of PPD among women from February 20th - March 30th (early wave of the COVID-19 epidemic in Europe) with those from early autumn 2019 (October 1st - November 10th, 2019). We chose this period because we decided that it is long enough before Christmas preparation time which can contribute to an intensification of symptoms that are compounded by Christmas-related stress - in all people, not just among risk groups.

### Procedure

Most European countries conduct screenings to monitor the mental state of women in the postpartum period. In Poland, such an obligation was introduced recently - in January 2019 when a new, national standard of a perinatal care was introduced. This standard organized, among others, procedures related to early support of women’s mental health after delivery. On this basis, the Polish Ministry of Health implemented regional programs aimed at screening postpartum mental health disorders and offering quick support in the place of a woman’s residence. The regional division makes it easier to coordinate the project by regional implementers and provide direct assistance to women from various places in Poland (not just large urban agglomerations). Regional programs are financed from public funds and co-financed by the European society’s funds. One of them is a project ‘Next Stop: Mum’ (no. POWR.05.01.00-00-0023/18), which was implemented in the northern macro-region of Poland by the Copernicus Health Entity along with the Institute of Psychology at the University of Gdansk in mid-2019. In addition to screening procedures implemented at hospitals and health facilities, implementers of the ‘Next Stop: Mum’ built an on-line platform for women seeking mental health support in the postpartum period. With this on-line formula women seeking postpartum support can independently and anonymously self-assess the severity of postpartum depression symptoms and quickly receive feedback. If a disturbingly high result is obtained, a woman is allowed to take advantage of free-of-cost psychological consultations at the place of her residence or online.

### Method

Women via on-line self-assessment are screened with the Edinburgh Postnatal Depression Scale (EPDS) which is a short (10-item), self-reporting tool that was designed by Cox et al. [27] to assist health professionals in detecting symptoms of PPD. The maximum score to be obtained in this scale is 30 points and the more points a woman receives, the more severe the depression symptoms can be suspected. Two cut-off points for EPDS are the most commonly used: 10–11 points are interpreted as slightly increased severity of PPD symptoms, whereas 12 or more points indicate a significant increase in PPD symptoms (requiring extended clinical examination). It is also worth adding that in some cases, a qualitative assessment of the patient’s response is made, or selected EPDS items, such as those related to thoughts about harming themselves, are discussed and analysed with the patient in detail. This tool is commonly used and recommended as a screening method when used by medical personnel who does not directly provide psychological/psychiatric services. However, a high score in the EPDS is an important reason to refer a woman for an extended clinical assessment. Training in the use of the EPDS scale is not compulsory but is often recommended when used by medical personnel who do not assess the severity of symptoms of psychiatric disorders daily.

### Ethical consideration

The protocol of this study was approved by the Ethics Board for Research Projects at the Institute of Psychology, University of Gdansk, Poland (decision no. 20/2019). The ethics committee indicated that all adult patients have been deemed ethically and medically capable of consenting for their participation in the research presented in this manuscript. The participants of the study were informed that by completing the electronic version of the EPDS questionnaire, they also consented to participate in the study. The Ethics Board for Research Projects (ethics committee) approved this procedure in above-mentioned decision no. 20/2019.

### Participants

The study analyzed data from 139 women who completed EPDS questionnaires on our on-line platform (‘Next Stop: Mum’) in the two time periods:

- 61 participants completed the EPDS questionnaire during the non-epidemic period: October 1st - November 10th, 2019,
- 78 participants completed the EPDS questionnaire during the COVID-19 epidemic period: February 20th - March 30th, 2020.

### Statistical analysis

We used SPSS Version 26.0 for statistical analyses. Intergroup differences in sociodemographic and biomedical variables were tested using the t-test (for quantitative variables) and chi-squared test (for qualitative variables). To compare the results in EPDS between the two groups, we used the independent t*-*test. The comparative analysis was preceded by Kolmogorov-Smirnov test (testing the normality of the EPDS results’ distribution). Finally, we described the frequencies of clinical results (regarding the cut-off points described above) among both groups.

## Results

### Biomedical and sociodemographic differences of the groups

Biomedical and sociodemographic characteristics of the participants are presented in Table 1.

**Table 1:** Biomedical and sociodemographic characteristics of the participants

Variable	Non-epidemic period: October 1st - November 10th (*n* = 61)	Early COVID-19 epidemic period: February 20th - March 30th, 2020 (*n* = 78)	*t test*	*p* value	Cohen’s *d*	Observed power
Woman age, years (mean ± SD)	31.04 ± 3.70	31.74 ± 5.06	−.86	.391	.16	.15
Baby gestational age during delivery, wk. (mean ± SD)	38.56 ± 3.55	38.88 ± 1.88	−.58	.563	.11	.09
Baby birth weight, g (mean ± SD)	3114.72 ± 1004.09	3233.03 ± 892.69	−.64	.524	.12	.11
Baby Apgar score – initial rating, n (mean ± SD)	9.31 ± 1.76	9.50 ± 1.49	−.59	.558	.12	.11
Baby Apgar score – final rating, n (mean ± SD)	9.61 ± .95	9.78 ± .92	−.92	.358	.18	.18
			*χ*^*2*^	*p* value	Cramér’s *V*	Observed power
Socioeconomic status (mean ± SD) ^a^	4.04 ± .63	3.73 ± .78	6.78	.079	.24	.57
Level of education (mean ± SD) ^b^	3.85 ± .36	3.80 ± .44	.85	.655	.09	.13
Occurence (%)						
Unthreatened pregnancy	45 (73.77%)	64 (82.05%)				
Vaginal delivery	36 (59.02%)	42 (53.84%)				
Baby born in a ‘good’ condition	55 (90.16%)	73 (93.59%)				
Complications after the delivery (relating to a mother)	6 (9.83%)	13 (16.70%)				
Complications after the delivery (relating to a baby)	11 (18.03%)	12 (15.38%)				

^a^ 1 – very bad, 2 – bad, 3 – average, 4 – good, 5 – very good^b^ 1 – primary education, 2 – vocational school, 3 – secondary education, 4 – higher education

All participants were women and groups did not differ in terms of any analysed sociodemographic and biomedical variables. Moreover, among women who participated in the study during the non-pandemic period (October 1st - November 10th, 2019) 58.82% were the residents of big cities (over 250,000 inhabitants), 11.74% lived in medium-sized cities (between 50 and 250,000 inhabitants), 9.80% lived in small cities (up to 50,000 inhabitants) and 19.60% lived in the countryside. Whereas, among women who participated in the study during the COVID-19 epidemic period (February 20th - March 30th, 2020) 47.69% were the residents of big cities (over 250,000 inhabitants), 23.07% lived in medium-sized cities (between 50 and 250,000 inhabitants), 6.15% lived in small cities (up to 50,000 inhabitants) and 23.07% lived in the countryside.

### The severity of the postpartum depression symptoms

**Fig. 1:**
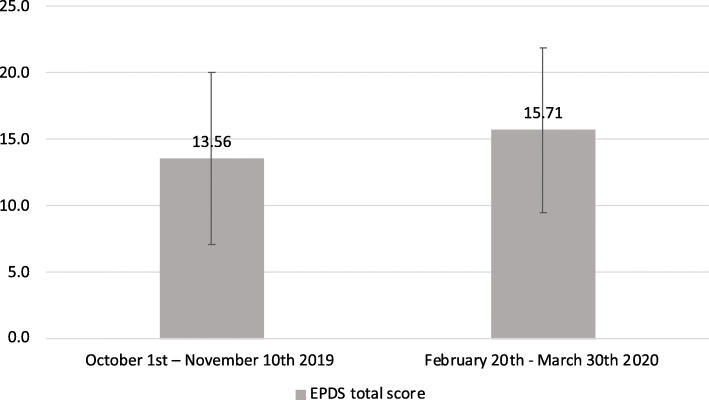
Severity of the postpartum depression symptoms during the neutral ‘non-epidemic’ and the COVID-19 epidemic period

The Kolmogorov–Smirnov test revealed that the distributions of the overall scores in EPDS were not significantly different from the normal distribution for both groups: from neutral ‘non-epidemic’ period: D(61) = 0.10; *p* = 0.20 and from the beginning of the COVID-19 epidemic: D(78) = 0.09; *p* = 0.20. Therefore, a decision was made to use the parametric test in further analysis. Independent t-test indicated on a significant difference (t = − 1.984; *p* = 0.025; Cohen’s d = 0.34; observed power = 0.63) in the overall scores of the postpartum depression symptoms among women who filled-in the on-line form in the period of February 20th - March 30th, 2020 (M = 15.71; SD = 6.23; *n* = 78) compared to the measurement from the neutral period of the October 1st – November 10th, 2019 (M = 13.56; SD = 6.46; *n* = 61). The details are presented on Fig. 1.

The distribution of results was than analysed in terms of their significance for subsequent expanded clinical diagnosis (see the details in Table 2).

**Table 2:** Occurrence of the clinical results in the EPDS assessments (regarding the cut-off points)

Result of the EPDS assessment	Non-epidemic period: October 1st - November 10th (*n* = 61)	Early COVID-19 epidemic period: February 20th - March 30th, 2020 (*n* = 78)
Normal range (0–9 points)	21.31%	16.67%
Slightly increased (10–11 points)	13.11%	8.97%
Increased (12 points and more)	65.57%	74.35%

## Discussion

This study investigated the severity of postpartum depression symptoms among women seeking mental health support at the beginning of the COVID-19 epidemic crisis in Poland. We assumed that the postpartum period is a particularly difficult moment (among others because of the psychological transition to the new role - a parent), which is often associated with mood disorders, and the beginning of the COVID-19 epidemic might have been an additional risk factor for the severity of postpartum depressive symptoms in women. Intrigued by the increased demand for mental health support from recipients of the project ‘Next Stop: Mum’ we decided to verify whether women seeking support at the beginning of the epidemic received higher result in the screening assessment of the PPD symptoms than women seeking support at neutral (non-epidemic) period. Our short study revealed a significantly greater severity of PPD symptoms among the first-mentioned group. This observation is consistent with earlier reports [9, 10] that indicated greater severity of depression symptoms among pregnant women during the COVID-19 epidemic. It can therefore be concluded that the epidemic crisis is associated with the intensification of mental health difficulties throughout the whole perinatal period (pregnancy and postpartum).

We assumed that concerns about the exposure to COVID-19 along with the government’s recommendations of a social distance, the experience of isolation, stressors of financial destabilization, can exacerbate depression symptoms in new mothers. The severity of depressive symptoms may also be associated with limited access to support sources, such as primary healthcare, lactation clinic. The lack of support in breastfeeding problems could lead to early breastfeeding cessation what also influences maternal mood. For example, according to Davey and colleagues [28] failure in breastfeeding was associated with postpartum depressive symptoms. Ystrom [29] indicated that women with high levels of anxiety and depression during pregnancy who stop breastfeeding early are at additional multiplicative risk for postpartum anxiety and depression. Exclusive breastfeeding is also a protective factor for mood disorders: Figueiredo et al. [30] observed a significant decrease in depression scores from childbirth to 3 months postpartum in women who maintained exclusive breastfeeding for greater than or equal to 3 months. The radical change in midwifery care in COVID–19 pandemic significantly deprived new mothers of usual care – in Poland new mothers benefit from six midwife homes visits, where the lactation support is usually offered. What is also important, although 116 (89%) countries reported that mental health and psychological support were part of their national COVID-19 response plans, only 17% said they had committed additional funding for this [31]. We assume that there is a need for low cost and effective recommendations for midwives and physicians to support mental health in pregnant and postpartum women. Based on the literature review, we prepared recommendations for medical staff, women, and their families, as well as the policy which can be divided into immediate actions as well as long-term strategic programs:

1. In the lack of / limited number of postpartum midwifery patronage visits, it is important to inform about perinatal prevention of mental health during woman’s hospital stay, e.g. by including information on the importance of monitoring the severity of depression symptoms after delivery on the discharge form. It is also important to inform about the local support system i.e. support groups in social networks for women in the postpartum period, safe forms (e.g. remote) of psychiatric and/or psychological consultation during an epidemic.
2. It is also important for medical staff to be able to inform mothers about the prevalence of postpartum depression in order to minimize fear and social stigma associated with postpartum emotional difficulties as well as pointing out that caring for mental health in the postpartum period may be particularly important during an epidemic crisis. Women may not be willing to provide information about their mental state during their stay in the maternity ward (e.g. due to fears of social stigma or possible consequences in the form of a “separation” from the child if such symptoms are revealed). It is also worth noting that many women are not willing to seek help in medical facilities after delivery because of concerns about the exposure of themselves or their child to COVID-19. In such situations, it is worth providing a link to an internet platform where new mothers can test the severity of depressive symptoms and receive guides on how to take care of postpartum mental health (point 4 below). Prevention and early intervention are the basis for effective detection of mental illness and thus it is worth considering the most effective ways of reaching people at high risk who need mental health support during an epidemic.
3. Despite the significantly limited availability of services during an epidemic crisis, online resources and treatment via telemedicine are gaining popularity. It has been proven so far that psychological online services are nearly as effective as direct assistance [32, 33]. Therefore, it is worth encouraging women to contact professionals - even during a pandemic - when they notice disturbing symptoms. It may be helpful to ensure woman that the meetings can take place in a safe formula e.g. via on-line / telephone consultation or with extreme caution for direct visits. Ensuring women with possible and safe ways of support can reduce their anxiety. Also, breastfeeding consultations can be conducted “online” – help in breastfeeding problems is also an important factor influencing postpartum mental health [34].
4. Many women give up psychiatric consultations during pregnancy and after delivery due to concerns about the rapid impact of antidepressants on the fetal / child. This also applies to women who undergo chronic treatment, and who - in case of pregnancy - often discontinue medication without psychiatric consultation. Thus, it seems important to promote varied treatment methods for perinatal psychiatric disorders and indicate the actual effects of using medications during pregnancy and in the postpartum period [35]. It also seems important to inform women about seeking information in reliable sources (e.g. during psychiatric/psychological consultations) not in unreliable sources e.g. random websites, Internet forums. This seems particularly important during the epidemic when women in the postpartum period often limit visits to medical facilities only to those which are obligatory.
5. Increased depressive symptoms may be a part of an adaptive response to extraordinary stress and psychotherapy techniques such as those based on the stress-adaptation model might be helpful. Women in the postpartum period can also benefit from some lifestyle changes focused on the daily routine adapted to the new pandemic reality. Such a self-care daily program can be based on the NEST-S principles: Nutrition, Exercise, Sleep, Time for Self, Support [36], in the form in which it enables mothers the care of a newborn baby. The dissemination of knowledge about NEST–S principles is important and there is still much to be done: for example, research shows [37] that most of the pregnant women are reluctant to exercise or only lightly active, what can also last in the postpartum period. Physical activity is a relatively costless intervention that can improve maternal wellbeing [37, 38]. Yet, for those women presenting with more severe mental health problems psychiatric treatments should be provided.
6. Lockdown in many cases can create new possibilities of co-parenting: working from home and social distancing can enable both partners to engage in childcare: fathers working from home can have more opportunities to support women emotionally – and that can be an important factor in adjusting to motherhood [39]. Additionally, partners who stay at home during the day also have a greater opportunity to observe changes in the mental state of woman, talk about it, and seek help or support outside. Thus, it is also worth to allocate some space on mental health support platforms for women where information for fathers (and other family members) about methods of supporting women in the postpartum during epidemic would be included.
7. According to Xiang, Yang and Li [40] in any biological disaster, themes of fear, uncertainty, and loss are common and may act as barriers to appropriate medical and mental health interventions. Access to mental health assessment, and the availability of support, treatment, and services are crucial during an epidemic crisis. Due to the possible negative consequences of postpartum depression for both the mother and her child, a woman needs to receive immediate help/support. Thus, it is worth creating a quick path for access to professionals for women in the postpartum period who struggle with symptoms of mental health disease e.g. postpartum depression. The implementation of treatment at an early stage of symptom development may shorten the time of therapy and allow women to fully enjoy motherhood, even in such difficult circumstances as the COVID-19 epidemic.
8. We believe that in the current epidemic situation, a very important element of psychological / psychiatric consultation is to support women in the possibility of thinking about painful emotions inscribed in this particularly difficult transition to motherhood. In practice, this means above all listening carefully to patients who often do not have the opportunity to freely share their experiences deviating from social expectations: doubts about their own competences in protecting the child and themselves from exposure to the virus, the experience of losing important moments of early motherhood due to restrictions associated with a pandemic (limited mobility, leaving home, meetings with family and loved ones). Concerns about subsequent months and a lack of predictability can increase mothers’ sense of stress. When women are unable to talk (and perhaps think) about such feelings, professionals can support this skill by asking questions about the type of experience (of loss, stress, doubts) emphasizing their normality in such a situation. In this way professionals can support the mentalizing skills of patients in thinking about experienced losses and the complexity of feelings inscribed in early motherhood.

## Conclusions

Our research indicates that during the epidemic crisis, women in the postpartum period may be in a greater need for mental health support. This is demonstrated by the increased severity of depressive symptoms among women seeking support during epidemic crisis compared to the neutral (pre-epidemic) period. Therefore, it seems important to design solutions for the increased emotional needs of women in the perinatal period in the case of the probable second wave of COVID-19.

### Limitations

A significant limitation of the presented study is its retrospective type, which made it impossible to design more complex model consisting of many additional variables. This short report aimed to provide early information about our observation indicating a higher severity of depressive symptoms during a pandemic among women seeking psychological help in our postpartum support program ‘Next Stop: Mum’. Since it is not a prospective study we did not plan to analyze multiple variables and build any models. However, this issue is worth considering in further research.

Another important limitation of this report is the relatively low power of the observed differences. Therefore, one should be careful in drawing conclusions and generalizing the observations from the study on population.

## Supplementary Information

**Additional file 1.** EPDS questionnaireR3

## Data Availability

The datasets used and/or analysed during the current study are available from the corresponding author on reasonable request.

## Abbreviations

SARS: Severe acute respiratory syndrome
EPDS: Edinburgh postnatal depression scale
PPD: Postpartum depression
COVID-19: Coronavirus disease 2019

## References

[1] Hamer K, Baran M, Marchlewska M. Czego boją się Polacy w związku z koronawirusem? [what are poles afraid of in connection with coronavirus ?]. 2020. https://covid19psychologiacom.files.wordpress.com/2020/03/koronawirus-raport-1-czego-bojacca8-siecca8-polacy.pdf. Accessed 10 July 2020.

[2] Holmes EA, O'Connor RC, Perry VH, Tracey I, Wessely S, Arseneault L (2020). Multidisciplinary research priorities for the COVID-19 pandemic: a call for action for mental health science. Lancet Psychiatry.

[3] Chen Q, Liang M, Li Y, Guo J, Fei D, Wang L (2020). Mental health care for medical staff in China during the COVID-19 outbreak. Lancet Psychiatry.

[4] Xiang YT, Yang Y, Li W, Zhang L, Zhang Q, Cheung T, Ng CH. Timely mental health care for the 2019 novel coronavirus outbreak is urgently needed. Lancet Psychiatry. 2020;7(3):228–9. 10.1016/S2215-0366(20)30046-8 PMC7128153 32032543

[5] Maunder R, Hunter J, Vincent L, Bennett J, Peladeau N, Leszcz M (2003). The immediate psychological and occupational impact of the 2003 SARS outbreak in a teaching hospital. CMAJ.

[6] Liu TB, Chen XY, Miao GD (2003). Recommendations on diagnostic criteria and prevention of SARS-related mental disorders. J Clin Psychol Med.

[7] Pfefferbaum B, North CS. Mental Health and the Covid-19 Pandemic. N Engl J Med. 2020;383(6):510–2. 10.1056/NEJMp2008017 32283003

[8] Hutchens BF, Kearney J. Risk factors for postpartum depression: an umbrella review. J Midwifery Womens Health 2020;66:1; doi:https://doi.org/10.1111/jmwh.13067. 10.1111/jmwh.13067 31970924

[9] Durankuş F, Aksu E. Effects of the COVID-19 pandemic on anxiety and depressive symptoms in pregnant women: a preliminary study. J Matern Fetal Neonatal Med. 2020:1–7. 10.1080/14767058.2020.1763946. 10.1080/14767058.2020.1763946 32419558

[10] Wu Y, Zhang C, Liu H, Duan C, Li C, Fan J, et al. Perinatal depressive and anxiety symptoms of pregnant women along with COVID-19 outbreak in China. Am J Obstet Gynecol. 2020. 10.1016/j.ajog.2020.05.009. 10.1016/j.ajog.2020.05.009 PMC7211756 32437665

[11] O'Hara MW, McCabe JE (2013). Postpartum depression: current status and future directions. Annu Rev Clin Psychol.

[12] Jaeschke RR, Dudek D, Topór-Mądry R, Drozdowicz K, Datka W, Siwek M (2017). Postpartum depression: bipolar or unipolar? Analysis of 434 polish postpartum women. Rev Bras Psiquiatr.

[13] Whiteford HA, Harris MG, McKeon G, Baxter A, Pennell C, Barendregt JJ (2013). Estimating remission from untreated major depression: a systematic review and meta-analysis. Psychol Med.

[14] O'Hara MW, Stuart S, Gorman LL, Wenzel A (2000). Efficacy of interpersonal psychotherapy for postpartum depression. Arch Gen Psychiatry.

[15] Goodman JH (2004). Postpartum depression beyond the early postpartum period. J Obstet Gynecol Neonatal Nurs.

[16] Wisner KL, Perel JM, Peindl KS, Hanusa BH (2004). Timing of depression recurrence in the first year after birth. J Affect Disord.

[17] Kendig S, Keats JP, Hoffman MC, Kay LB, Miller ES, Moore TA (2017). Consensus bundle on maternal mental health: perinatal depression and anxiety. Obstet Gynecol.

[18] Knight M, Kenyon S, Brocklehurst P, Neilson J, Shakespeare J, Kurinczuk JJ (2017). Saving lives, improving mothers' care: lessons learned to inform future maternity care from the UK and Ireland confidential enquiries into maternal deaths and morbidity 2009–2012.

[19] Wisner KL, Miller ES, Tandon D (2019). Attention to prevention-can we stop perinatal depression before it starts?. JAMA Psychiatry.

[20] Knight M, Tuffnell D, Kenyon S, Shakespeare J, Gray R, Kurinczuk JJ (2015). Saving lives, improving mothers' care: surveillance of maternal deaths in the UK 2011–13 and lessons learned to inform maternity care from the UK and Ireland. Confidential enquiries into maternal deaths and morbidity 2009–13.

[21] Chatterji P, Markowitz S (2012). Family leave after childbirth and the mental health of new mothers. J Ment Health Policy Econ.

[22] Kornfeind KR, Sipsma HL (2018). Exploring the link between maternity leave and postpartum depression. Womens Health Issues.

[23] Maliszewska K, Świątkowska-Freund M, Bidzan M, Preis K (2017). Screening for maternal postpartum depression and associations with personality traits and social support. A polish follow-up study 4 weeks and 3 months after delivery. Psychiatr Pol.

[24] Cowan K. Survey results: understanding people’s concerns about the mental health impacts of the COVID-19 pandemic. Acad Med Sci MQ Ment Health Report. 2020; https://acmedsci.ac.uk/file-download/99436893. Accessed June 17, 2020.

[25] Matthews T, Danese A, Caspi A, Fisher HL, Goldman-Mellor S, Kepa A (2019). Lonely young adults in modern Britain: findings from an epidemiological cohort study. Psychol Med.

[26] O'Connor RC, Kirtley OJ (2018). The integrated motivational-volitional model of suicidal behaviour. Philos Trans R Soc Lond B Biol Sci.

[27] Cox JL, Holden JM, Sagovsky R (1987). Detection of postnatal depression. Development of the 10-item Edinburgh postnatal depression scale. Br J Psychiatry.

[28] Davey HL, Tough SC, Adair CE, Benzies KM (2011). Risk factors for sub-clinical and major postpartum depression among a community cohort of Canadian women. Matern Child Health J.

[29] Ystrom E. Breastfeeding cessation and symptoms of anxiety and depression: a longitudinal cohort study. BMC Pregnancy Childbirth 2012; 12: 36. https://doi.org/10.1186/1471-2393-12-36. 10.1186/1471-2393-12-36 PMC3449190 22621668

[30] Figueiredo B, Canário C, Field T (2014). Breastfeeding is negatively affected by prenatal depression and reduces postpartum depression. Psychol Med.

[31] The Lancet Infectious Diseases Editorial. The intersection of COVID-19 and mental health. Lancet Infect Dis. 2020;20(11):1217. 10.1016/S1473-3099(20)30797-0. 10.1016/S1473-3099(20)30797-0 PMC7544473 33038942

[32] American Psychological Association (2013). Guidelines for the practice of telepsychology. The American psychologist.

[33] Scharff JS (2013). Psychoanalysis online : mental health, Teletherapy, and training.

[34] Chrzan-Dętkoś M, Pietkiewicz A, Żołnowska J, Pizuńska D (2020). The program of psychological and breastfeeding support "Maternity step by step": an example of effective solution for the prevention, diagnostics and treatment of prenatal and postpartum depression. Psychiatria Pol.

[35] Samochowiec J, Rybakowski J, Galecki P, Szulc A, Rymaszewska J, Cubala WJ (2019). Recommendations of the polish psychiatric association for treatment of affective disorders in women of childbearing age. Part I: treatment of depression. Psychiatr Pol.

[36] Haring M, Smith JE, Bodnar D, Misri S, Ryan D (2013). Coping with anxiety during pregnancy and following the birth: a cognitive behaviour therapybased self-management-guide for women and health care providers.

[37] Rabiepoor S, Rezavand S, Yas A, Ghanizadeh N (2019). Influential factors in physical activity amongst pregnant women. Balt J Health Phys Activ.

[38] Zerf M (2019). Effects of walking training performed using continuous and interval methods on weight loss as effective strategies among postpartum women. Balt J Health Phys Activ.

[39] Kaźmierczak M, Kiełbratowska B, Karasiewicz K (2015). The other side of the mirror – the role of partner’s empathy in transition to parenthood. Health Psychol Rep.

[40] Xiang YT, Yang Y, Li W, Zhang L, Zhang Q, Cheung T (2020). Timely mental health care for the 2019 novel coronavirus outbreak is urgently needed. Lancet Psychiatry.

