# Supplementary material for: The need for additional mental health support for women in the postpartum period in the times of epidemic crisis
Source: BMC Pregnancy Childbirth. 2021 Feb 8;21:114. doi: 10.1186/s12884-021-03544-8 (PMC7869073; doi:10.1186/s12884-021-03544-8)
Supplement: Supplementary file 1 — Additional file 1. EPDS questionnaireR3 [file 12884_2021_3544_MOESM1_ESM.docx]

**Edinburgh Postnatal Depression Scale (EPDS)^1^**

*Please check the answer that comes closest to how you have felt IN THE PAST 7 DAYS, not just how you feel today.*

*Here is an example, already completed.*

*I have felt happy:*

- *
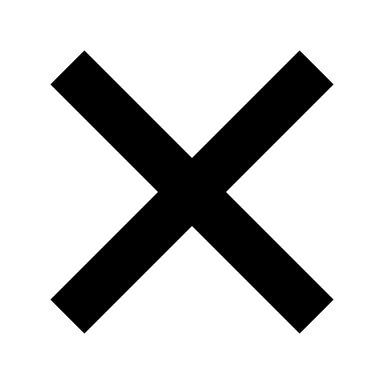
Yes, all the time*
- *Yes, most of the time*
- *No, not very often*
- *No, not at all*

*This would mean: “I have felt happy most of the time” during the past week. Please complete the other questions in the same way.*

In the past 7 days:

1. I have been able to laugh and see the funny side of things

- As much as I always could
- Not quite so much now
- Definitely not so much now
- Not at all

2. I have looked forward with enjoyment to things

- As much as I ever did
- Rather less than I used to
- Definitely less than I used to
- Hardly at all

3. I have blamed myself unnecessarily when things went wrong

- Yes, most of the time
- Yes, some of the time
- Not very often
- No, never

4. I have been anxious or worried for no good reason

- No, not at all
- Hardly ever
- Yes, sometimes
- Yes, very often

5 I have felt scared or panicky for no very good reason

- Yes, quite a lot
- Yes, sometimes
- No, not much
- No, not at all

6. Things have been getting on top of me

- Yes, most of the time I haven’t been able to cope at all
- Yes, sometimes I haven’t been coping as well as usual
- No, most of the time I have coped quite well
- No, I have been coping as well as ever

7. I have been so unhappy that I have had difficulty sleeping

- Yes, most of the time
- Yes, sometimes
- Not very often
- No, not at all

8. I have felt sad or miserable

- Yes, most of the time
- Yes, quite often
- Not very often
- No, not at all

9. I have been so unhappy that I have been crying

- Yes, most of the time
- Yes, quite often
- Only occasionally
- No, never

10. The thought of harming myself has occurred to me

- Yes, quite often
- Sometimes
- Hardly ever
- Never

^1^Source: Cox, J.L., Holden, J.M., and Sagovsky, R. 1987. Detection of postnatal depression: Development of the 10-item Edinburgh Postnatal Depression Scale. *British Journal of Psychiatry* 150:782-786
